# Survive and Thrive in Brazil: estimating the impact of the Boa Vista early childhood program

**DOI:** 10.1093/heapol/czag057

**Published:** 2026-04-27

**Authors:** Alexandra Brentani, Georg Loss, Luana Bessa, Susan Chang-Lopez, Susan P Walker, Ana Paula Ferrer, Sandra Grisi, Florencia Lopez Boo, Günther Fink

**Affiliations:** Department of Pediatrics, University of São Paulo Medical School FMUSP, Av. Dr. Enéas de Carvalho Aguiar, 647, 05403-000, Sao Paulo, Brazil; Child Development Center, University of São Paulo Medical School CEDI-FMUSP, Av. Dr. Enéas de Carvalho Aguiar, 647, 04503-000, Sao Paulo, Brazil; Department of Economics, University of Basel, Kreuzstrasse 2, Allschwil 4213, Switzerland; Department of Research, Swiss Tropical and Public Health Institute, Kreuzstrasse 2, Allschwil 4123, Switzerland; Department of Pediatrics, University of São Paulo Medical School FMUSP, Av. Dr. Enéas de Carvalho Aguiar, 647, 05403-000, Sao Paulo, Brazil; Child Development Center, University of São Paulo Medical School CEDI-FMUSP, Av. Dr. Enéas de Carvalho Aguiar, 647, 04503-000, Sao Paulo, Brazil; Caribbean Institute for Health Research, The University of the West Indies, Mona Campus, Kingston 7, Jamaica; Caribbean Institute for Health Research, The University of the West Indies, Mona Campus, Kingston 7, Jamaica; Department of Pediatrics, University of São Paulo Medical School FMUSP, Av. Dr. Enéas de Carvalho Aguiar, 647, 05403-000, Sao Paulo, Brazil; Child Development Center, University of São Paulo Medical School CEDI-FMUSP, Av. Dr. Enéas de Carvalho Aguiar, 647, 04503-000, Sao Paulo, Brazil; Department of Pediatrics, University of São Paulo Medical School FMUSP, Av. Dr. Enéas de Carvalho Aguiar, 647, 05403-000, Sao Paulo, Brazil; Child Development Center, University of São Paulo Medical School CEDI-FMUSP, Av. Dr. Enéas de Carvalho Aguiar, 647, 04503-000, Sao Paulo, Brazil; Steinhardt School of Culture, Education, and Human Development, New York University, 246 Greene Street, 615E, New York, NY 100037, United States; Global TIES for Children, New York University, 246 Greene Street, 615E, New York, NY 100037, United States; Department of Economics, University of Basel, Kreuzstrasse 2, Allschwil 4213, Switzerland; Department of Research, Swiss Tropical and Public Health Institute, Kreuzstrasse 2, Allschwil 4123, Switzerland

**Keywords:** impact evaluation, home visit and group parenting programs, early childhood development, neonatal mortality

## Abstract

This study aimed to evaluate the impact of the Survive and Thrive program on neonatal mortality and child development in Boa Vista, Brazil. Between November 2017 and May 2021, the program to support pregnant women as well as mothers of children under age 3 was rolled out in three phases. Neighborhoods were selected either for home visits or for group meetings held at Social Assistance Reference Centers. To allow for an evaluation of the program, the timing of the rollout was randomized at the neighborhood level. To assess the impact of the program on neonatal mortality, we used complete vital registration data from the period 2010 to 2010, and estimated differences in child mortality before and after the program were launched. Impact on child development was assessed through a detailed assessment of 744 children born in 2019 using the Caregiver Reported Early Child Development Index and Regional Project on Child Development Indicators instruments. Home visits resulted in a significant improvement in child development [*d* = 0.28, 95% Confidence Interval (CI) (−0.013, 0.57), *P*-value of .06] and reduction of neonatal mortality [Risk Ratio (RR) 0.58, 95% CI (0.36, 0.93), *P*-value of .02]. No impacts were found for the group meetings. The findings indicate that a home visiting program beginning in pregnancy can significantly reduce neonatal mortality and improve child development in poor urban neighborhoods of Brazil.

Key messages

- What is already known on this topic: home visit programs to promote early childhood development are widely recognized as one of the most effective strategies to support vulnerable populations.
- What this study adds: in this study, we adapted a home visit curriculum to begin during pregnancy and continue through 36 months, designed for large-scale implementation in Brazil. While home visits yielded significant improvement in neonatal mortality and child development, center-based group meetings did not have any impact on either outcome.
- How this study might affect research, practice, or policy: providing home visits during pregnancy can significantly reduce neonatal mortality. Furthermore, the adapted curriculum can be integrated into large-scale policies, such as Brazil’s national early childhood development program, Criança Feliz.

## Introduction

Neonatal mortality remains a critical global health challenge and a key indicator of the overall well-being of populations. Despite major international efforts, an estimated 2.4 million neonatal deaths occur annually, accounting for nearly 47% of all under-five deaths (Lawn et al. 2016). Neonatal mortality disproportionately affects marginalized populations and communities with often limited access to healthcare, education, and other resources (Bhutta et al. 2014).

Sustainable Development Goal 3 aims to ensure healthy lives and promote well-being for all, with a specific target of reducing neonatal mortality to fewer than 12 deaths per 1000 live births by 2030 (United Nations 2015).

Public policies play a pivotal role in shaping the health trajectory of populations, particularly in developing countries where neonatal mortality and early childhood development remain pressing challenges. Public policies that ensure a continuum of care supporting children from birth through early childhood are thus of high priority. In the realm of maternal and child health, home visit (HV) programs are widely recognized as one of the most effective strategies to support vulnerable populations. The focus of such programs extends beyond conventional healthcare settings, reaching into the homes of expectant mothers and newborns (Baqui 2009, Neupane et al. 2017, Kante et al. 2019).

In addition, a large body of literature underscores the importance of early childhood experiences in shaping brain architecture and influencing early development (Black et al. 2017), with life-long implications for academic achievement, socioemotional functioning, and overall health (Shonkoff 2016). Positive early experiences, characterized by responsive caregiving, enriching learning environments, and secure attachments, have been associated with enhanced cognitive abilities, better academic achievement, and improved socioemotional well-being (Jeong et al. 2021, Jervis et al. 2023). Conversely, adverse early experiences can disrupt healthy brain development and contribute to long-term negative outcomes, including cognitive impairments and increased vulnerability to mental health disorders (National Scientific Council on the Developing Child 2005/2014). Poverty and lack of social support can result in increased exposure to biological and psychosocial risks that negatively affect brain structure and function and trigger adverse behavioral adaptations. As risks accumulate, development is increasingly compromised (Walker et al. 2007). Globally, an estimated 250 million children under 5 years (43%) are at risk of not achieving their developmental potential in the earliest years of life due to nutritional, health, and psychosocial risks (Grantham-McGregor et al. 2007). The profound impact of early experiences underscores the plasticity of the developing brain as well as the importance of interventions to support children during this stage. A recent review by the Inter-American Development Bank shows that despite policy and programmatic progress in Latin America and Brazil, gaps in Early Child Development (ECD) levels by socioeconomic status and ethnicity emerge very early in life, and by primary school-age, they are substantial and can increase inequalities later in life (Inter-American Development Bank 2024).

HV programs can contribute to early childhood development by offering support in promoting positive caregiving practices, responsive parenting, and early learning activities (Sweet and Appelbaum 2004, Yousafzai and Aboud 2014 , Jeong et al. 2021). Among early childhood home visiting and parenting programs, the “Reach Up” early childhood parenting program has been effective across varying global contexts in fostering cognitive and socioemotional development of young children (Brentani et al. 2021, Jervis et al. 2023). The program promotes parent skills in responsive caregiving and early learning and can be combined with health and nutrition to create a comprehensive framework that addresses the multifaceted needs of young children (Grantham-McGregor et al. 1991, Black et al. 2023). Various elements of the Reach Up curriculum, training, and supervision packages have been used in Latin American Countries (LAC) countries, such as in Ecuador, Nicaragua, Peru and Fortaleza, Brazil. Rigorous evaluations of other at-scale home visiting models in LAC offer mixed findings, but show that home visiting models, when successful, offer high benefit–cost ratios (Inter-American Development Bank 2024).

The objective of the Survive and Thrive program was to promote child survival and development in Boa Vista, Brazil, through the rollout of a home or center-based (CB) parenting intervention building on the Reach Up package. The objective of the evaluation study reported here was to assess the impact of both home visiting and the CB intervention on child survival and child development using administrative and survey data.

## Materials and methods

### Study design

**Figure 1:**
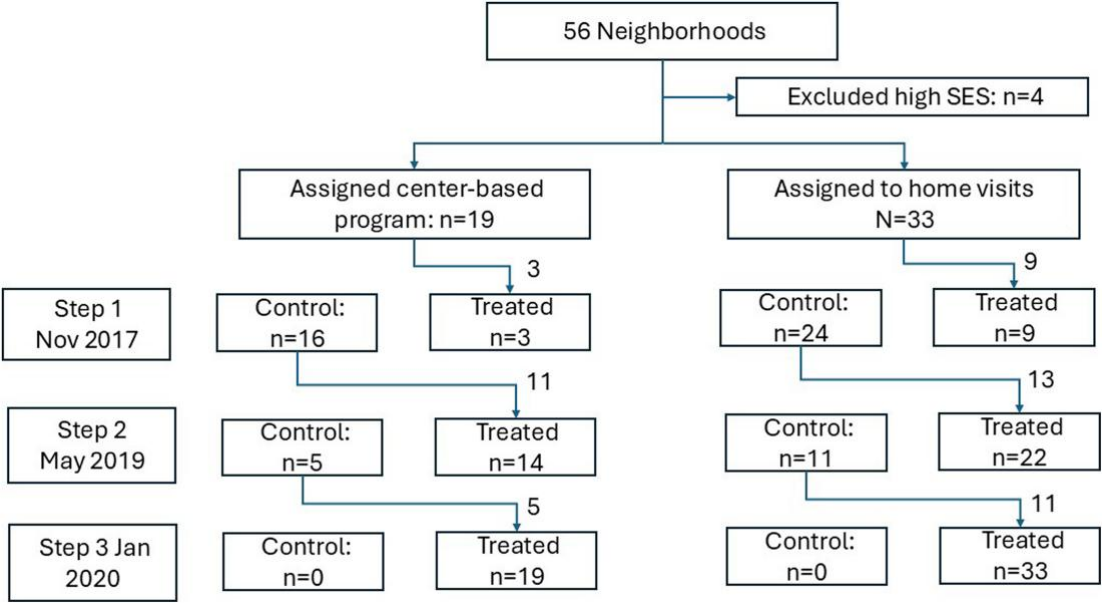
Study design.

This study explores the stepped-wedge cluster-randomized rollout of the program to assess the impact of the Survive and Thrive program in Boa Vista. Study clusters correspond to neighborhoods within the municipality of Boa Vista. Initially, clusters were randomly allocated to HV or CB group meetings or control. Figure 1 illustrates the overall study design. A total of 52 neighborhoods received ECD interventions; 33 were selected for HVs and 19 for CB group meetings. Within each modality, clusters were randomly assigned to begin receiving the program in three steps: in the first step, HVs were launched in nine neighborhoods and CB group meetings in three neighborhoods. In the second step, 13 neighborhoods were added to HV and 11 to CB group meetings. In the last step, programs were rolled out to all remaining neighborhoods. Study design and reporting followed the Consort Guidelines (Davey et al. 2025, Hemming et al. 2018).

To estimate the causal impact of the Survive and Thrive Program on child health and development, we applied two parallel strategies:

- First, to assess the impact on child survival, we used vital registration data on all children born in the municipality between 2010 and 2020. This data set covers ∼75 000 live births overall, and 11 000 live births in treated municipalities, allowing us to detect relatively small mortality differences in models controlling for year and neighborhood fixed effects (FEs) as well as neighborhood-specific (linear) time trends.
- Second, to assess the impact on child development, we assessed the development of ∼800 randomly selected children born in 2019 through trained field workers. As shown in Fig. 1, 2019 is the year with most program exposure variation in our sample: at the beginning of the year (Step 1), only 12 neighborhoods received interventions. At the end of the year (Step 2), the same was true for 36 neighborhoods, which means that we can explore random variations in program exposure both across neighborhoods and within neighborhoods (switchers).

### Study setting

In 2016, Brazil launched the ambitious Programa Criança Feliz (PCF), aiming to provide early childhood support to vulnerable families across Brazil. Program adoption is voluntary, and in 2017, a total of 3014 Brazilian municipalities were enrolled. Although the national program recommended the Care for Child Development model, exploring alternative packages was endorsed by the government (21). Funding from the national program was provided to municipalities interested in setting up ECD programs for vulnerable populations and thus also made available to the Survive and Thrive program evaluated here.

The study was conducted in Boa Vista, the capital of Roraima state, located in the Northern region of Brazil, ∼200 km from the Venezuelan border. Supplementary Fig. S1 shows the location of Roraima state as well as Boa Vista’s location within the state. As of 2017, Boa Vista’s estimated population was 303 020; 30% of this population was classified as poor (IBGE 2022). The municipality comprising 56 neighborhoods, was enrolled in the PCF in 2017 and started implementation using the adapted Reach Up curriculum. Forty-two neighborhoods were classified as having medium or high vulnerability and were targeted for program implementation (Supplementary Fig. S2). We used a stepped-wedge design to randomize the neighborhoods in the study arms. In Year 1, 12 neighborhoods (clusters) were allocated in the intervention group (three received CB group meetings and nine HVs), another 21 neighborhoods (clusters) were allocated in the intervention group (nine CB group meetings and 11 HVs) in Year 2, and in Year 3, the intervention was expanded to the remaining vulnerable neighborhoods. Outside of the study, the intervention was also offered for vulnerable communities residing in 14 nonvulnerable neighborhoods, in Year 3 (Supplementary Fig. S3a and b).

### Participants

Starting in 2017, pregnant women from 21 to 23 weeks of gestation enrolled in antenatal care or at the social assistance service were screened for inclusion and considered eligible if they met at least one of the following criteria: (i) received the national conditional cash transfer program (“Bolsa Família”) or were included in the “Bolsa Familia” waiting list, (ii) were under 19 years when they became pregnant, (iii) had declared been exposed to sexual or domestic violence, or (iv) declared a per capita income of less than a quarter of the minimum wage (BR$937.00/US$285.00 in 2017). Participants’ allocation was based on residential neighborhood. All participants signed an informed consent form prior to baseline data collection. Following the PCF guidelines, enrollment in the program is continuous for eligible families with pregnant women until child’s 36 months of age.

### Interventions

The intervention materials used were based on the Reach Up package previously adapted to Brazil in a São Paulo study (Brentani et al. 2021). Given the relatively high burden of neonatal mortality in the region, additional modules starting during the third trimester of gestation were added to the curriculum. In partnership with the Federal level of the PCF and the municipality of Boa Vista, Roraima, the adapted version of Reach Up was integrated in the municipality’s ECD program called “Programa Família que Acolhe.”

To support pregnant women and mothers of newborns, two intervention platforms were tested as part of this project: CB support groups meetings and one-to-one HVs.

#### Home visits

Families in the home visiting arm were visited every 2 weeks by a newly trained cadre of social visitors, professionals hired by the social assistance units with medium level of education who received specific training from the research team to perform the HVs. As already mentioned above, the curriculum used for these visits was based on the Reach Up program originally developed in Jamaica (Grantham-McGregor et al. 1991) and adapted to the Brazilian context and to the PCF. The curriculum previously evaluated in São Paulo was modified to include content for pregnant women during the third trimester of pregnancy and for children during the first 6 months. The pregnancy module offered information about the importance of antenatal care and breastfeeding and aimed to improve positive parenting practices, bonding, and attachment. Additionally, an alarm symptoms checklist was used every visit to identify immediate referral needs. The neonatal module involved three visits, in the first week, 15 days, and 28 days postpartum, focusing on maternal/child alarm symptoms, breastfeeding, and bonding. The child development module delivered from 1 to 36 months included three to four age-appropriate play and language activities per visit addressing different developmental domains (gross and fine motor, language, cognitive and social–emotional) and focusing on positive parenting practices and quality parent/child interactions. A child-related alarm symptoms checklist was used to identify referrals needs. Visits for pregnant women and children were conducted at 2-week intervals, lasted around 45 minutes, and caregivers were encouraged to engage children in the play activities between visits. For pregnant women, the visit included an action plan addressing a topic discussed during the preceding visit. All modules were delivered by trained social visitors aiming to support 30 families per visitor (60 visits per month). The municipality also hired a supervisor with a graduate degree and prior experience in social assistance for each social assistance unit. Their role was to supervise the work of the visitors, monitor program implementation, and detect training needs. The supervisors, together with the social service unit manager, were responsible for the referral of the participants to other social assistance services or healthcare services when needed.

#### Center-based group meetings

CB groups were set up in the seven social service unit [Social Assistance Reference Centers (CRAS)] buildings, which are centrally located and supposed to be easily reached by participants from the nearby neighborhoods from all vulnerable neighborhoods. The Reach Up curriculum was adapted for small group meetings held every 2 weeks and covered the same age-range topics as the Home Visit Curriculum and with the same objectives. The content of these topics matched the content of the HVs with facilitators working with groups of 6–10 pregnant mothers of similar gestational age and groups of mothers and children of similar child age. The CB curriculum offered only two activities per meeting due to the higher number of participants. Additionally, the meeting duration was longer (90 minutes) than the HV sessions (45 minutes). The neonatal module was limited to one to two sessions due to lower expected engagement during the first weeks of infants’ lives. Newly hired and trained moderators, with the same profile as the home visitors, ran three age-specific sessions daily. Participants missing three consecutive sessions were contacted by team supervisors for feedback and to resolve issues to continue participation. The supervisor from each social assistance unit was responsible for the group facilitators as well as the visitors.

Both home visitors and group meeting facilitators received 40 hours of training prior to deployment. All training was conducted by a certified Reach Up trainer. One supervisor, located in each CRAS, was responsible for 15 home visitors/facilitators. An additional 8-hour training course for the supervisors was conducted for all team supervisors, using the adapted version of the Reach Up supervisor manual.

### Time points

The first round of interventions (Step 1) was launched in November 2017. The second phase (Step 2) was launched in May 2019, and Step 3 was launched in January 2020.

### Monitoring and quality control

The implementation of visits and group meetings implementation was monitored monthly by the research team. Supervisors’ reports included information on the number of families enrolled, dropouts, and refusals. The visitors and group facilitators filled out a visit/group meeting log after each session comprising information about the participation, who was present, the quality of the interaction, and child’s ability to perform the activities. The supervisors observed a visit/group meeting of each visitor/facilitator on a monthly basis, filling out the Reach Up Observers checklist, a Reach Up–based instrument used to assess the quality and fidelity of the visits. Feedback was provided to the visitor immediately after the observed visit/group meeting to improve professionals’ skills (Rubio-Codina et al. 2019) A refreshment training was provided by the research group to all staff members in each year of the project.

### Primary and secondary study outcomes

The primary outcome was children’s overall development as measured by the Regional Project on Child Development Indicators (PRIDI) (Verdisco et al. 2015). Additional data on child development was collected using the Caregiver Reported Early Child Development Index (CREDI) (Altafim et al. 2020). The Strengths and Difficulties Questionnaire (Goodman 1997) was also used to assess children’s socioemotional development. Participants who were born in 2019 were assessed at endline, at ∼30 months of age. Endline assessments were performed by a previously trained group of independent assessors during a HV. The assessors were blinded to the participants’ allocation group. The secondary study outcome was neonatal mortality, defined as an infant death between Day 0 and Day 28 of a live born infant as reported in Brazil’s vital registration system described in further detail below. Similarly, early neonatal mortality was defined as an infant death between Day 0 and Day 7.

### Sample size and power calculations

The study was powered to detect an intention-to-treat effect of 0.25 SD for child development, with power 0.9 for each of the two intervention arms compared with the control arm, assuming an intraclass correlation of 0.02 and alpha = 0.05 with an endline sample of 30 children per cluster randomly selected from the larger study population.

For neonatal mortality, complete birth and mortality records were obtained from 2010 to 2020. With an initial neonatal mortality rate of 12 deaths per 1000 and a total anticipated sample size of 55 000 untreated children and 5000 treated children, the study was powered to detect a 35% reduction in neonatal mortality with probability .77 and a 40% reduction in neonatal mortality with probability .89.

### COVID-19

The SARS CoV 2 pandemic (COVI-19) started during the last step of the study randomization. All activities were suspended in early 2020, and face-to-face meetings were reestablished by late 2021; therefore, all the study participants received remote meetings delivered via WhatsApp during 2020 and 2021. Endline data collection was postponed to December 2021 and included only the children born in 2019, so that the participants in the intervention groups were exposed to at least 6 months of face-to-face meetings.

### Protocol deviations

In the original protocol, an endline sample of 3000 children was foreseen. Given budgetary constraints and COVID-19–related delays, we revised our field work plan for the child developmental assessments at endline, targeting a smaller sample size of ∼800 children born in 2019. This still provided us with sufficient power to detect a 0.3 SD difference in child developmental outcomes, which is within the range of impacts found in similar studies (Jeong et al. 2021).

### Randomization procedures

Randomization was based on a simple random number draw created in Stata, and neighborhoods with medium or high vulnerability according to the municipality’s social service vulnerability criteria were prioritized for earlier rollout. Neighborhoods were first randomized to an intervention group (HV, CB) and then to Steps 1–3. Neighborhoods where the intervention had not yet been rolled out served as controls. Supplementary Fig. S3a and b details the program and start date assigned to each neighborhood.

### Data

To assess the impact of the program on neonatal survival, we used data from Brazil’s vital registration system. All live births are recorded in Brazil’s Live Birth Information System (SINASC), and all deaths are recorded in Brazil’s Mortality Information System (SIM). We included data covering the period from 1 January 2010 to 31 December 2020. For deaths relevant to this analysis (i.e. neonatal deaths of live births), SINASC provided the following birth-related and demographic data for each live birth: birthday, mother’s name, maternal age, maternal education, maternal nationality, residential area, and delivery type. Information on mother and child ethnicities was limited and therefore not included in the analysis. During the study period, a total of 663 neonatal deaths were recorded in Boa Vista. Neonatal deaths were defined as deaths within the first 28 days. SIM further provided information on child’s birthday, mother’s name and residential area, and limited information on maternal age, maternal education, mother’s nationality, residential area, and delivery type. SIM neonatal death records and SINASC live birth records were merged, creating the analysis data set using the SINASC unique identifier (when available in both data sets) or probabilistic and manual matching (for more details, see Supplementary material). For variables with varying information in both databases, SINASC information was used for the analysis, as it reflected information collected closer to the time of birth. All analyses were restricted to Boa Vista neighborhoods participating in the Survive and Thrive study. Records not containing residential neighborhood information were omitted from the analysis set as this information was critical to treatment assignment.

Data on child development at endline was collected through one home-based interview and direct child assessments using the PRIDI tool. During the HV, a previously trained assessor would collect Social Economical Status (SES) information, parenting practices, home environment, and the parent reported child development CREDI scale. After that, during the same visit, the assessor administered the PRIDI scale directly with the child. Following the protocol adaptations due to the COVID-19 pandemic previously described, endline data collection was restricted to children born in 2019. As Fig. 1 illustrates, 2019 is the year with most program exposure variation in our sample: at the beginning of the year (Step 1), only 12 neighborhoods received interventions. At the end of the year (Step 2), the same was true for 36 neighborhoods, which means that we can explore random variations in program exposure both across and within neighborhoods (for those who switched from control to treatment in Step 2). Supplementary Table S1 shows the number of children treated and control conditions in each neighborhood.

### Variables

The primary child development outcome was the PRIDI scores, which we normalized to mean zero and SD 1 within our sample. We also analyzed CREDI and the Strengths and Difficulties Questionnaire (SDQ) scores as alternative caregiver-reported measures of child development and behavior, both normalized.

Variables used in the mortality analysis were maternal age, maternal education (none, 1–3 years, 4–7 years, 8–11years, 12 or more years), nationality (Brazilian vs. not), and delivery type (vaginal vs. C-section).

### Data analysis

Data were summarized using standard descriptive statistics. Birth and mortality trends were explored by aggregating data on annual level. The impact on child development was computed using PRIDI, CREDI, and SDQ total scores. Given that the stepped-wedge design resulted in both spatial and temporal variation in treatments, we analyzed developmental outcomes both with cluster-level random and cluster-level FEs. The random effect (RE) model is more efficient in principle (since it explores both cross-sectional and temporal variation) but can be biased if participant characteristics vary across neighborhoods. Our models were estimated using intent-to-treat estimates (ITT). The adjusted model included child age in months, caregiver age, caregiver education, marital status, cash transfer program receipt, and alcohol or tobacco use were used as covariates.

The impact on neonatal survival was estimated at the individual level for the period from 2010 to 2020 with intervention periods covering from November 2017 to December 2020. The inclusion of all children for the period 2010–20 allows us to fit neighborhood-specific time trends and identify deviations when interventions start. Children were coded as treated if interventions had been launched in their respective residential neighborhoods on the day of their birth. Given the gradual rollout of programs within each neighborhood, we divided treated periods into (i) the initial (partial) intervention phase covering the first 90 days since intervention start and (ii) the main intervention phase: from 91 days after start until the end of the mortality analysis period, i.e. December 2020.

Generalized linear regression models with neighborhood clustered standard errors were used to estimate the ITT effects of treatments on neonatal mortality. The intervention variables were entered as dummies for each phase of partial and full treatment. The models were further adjusted for maternal age, maternal education, mother’s nationality, delivery type, and year. Given the low rates of missing data for covariates (<1%; see Supplementary Table S2), all regression models were computed using complete cases or a multiple imputation approach with chained equations (using the mi package in Stata with 50 imputed sets) to check robustness.

All analyses were done using STATA 16 SE (StataCorp, College Station, TX). A *P*-value of <.05 was considered statistically significant, and confidence intervals and adjusted and unadjusted estimates were reported for all results.

### Ethics and protocol

The data collected for this project was approved by the Ethical Committee of the authors’ institution.

## Results

**Figure 2:**
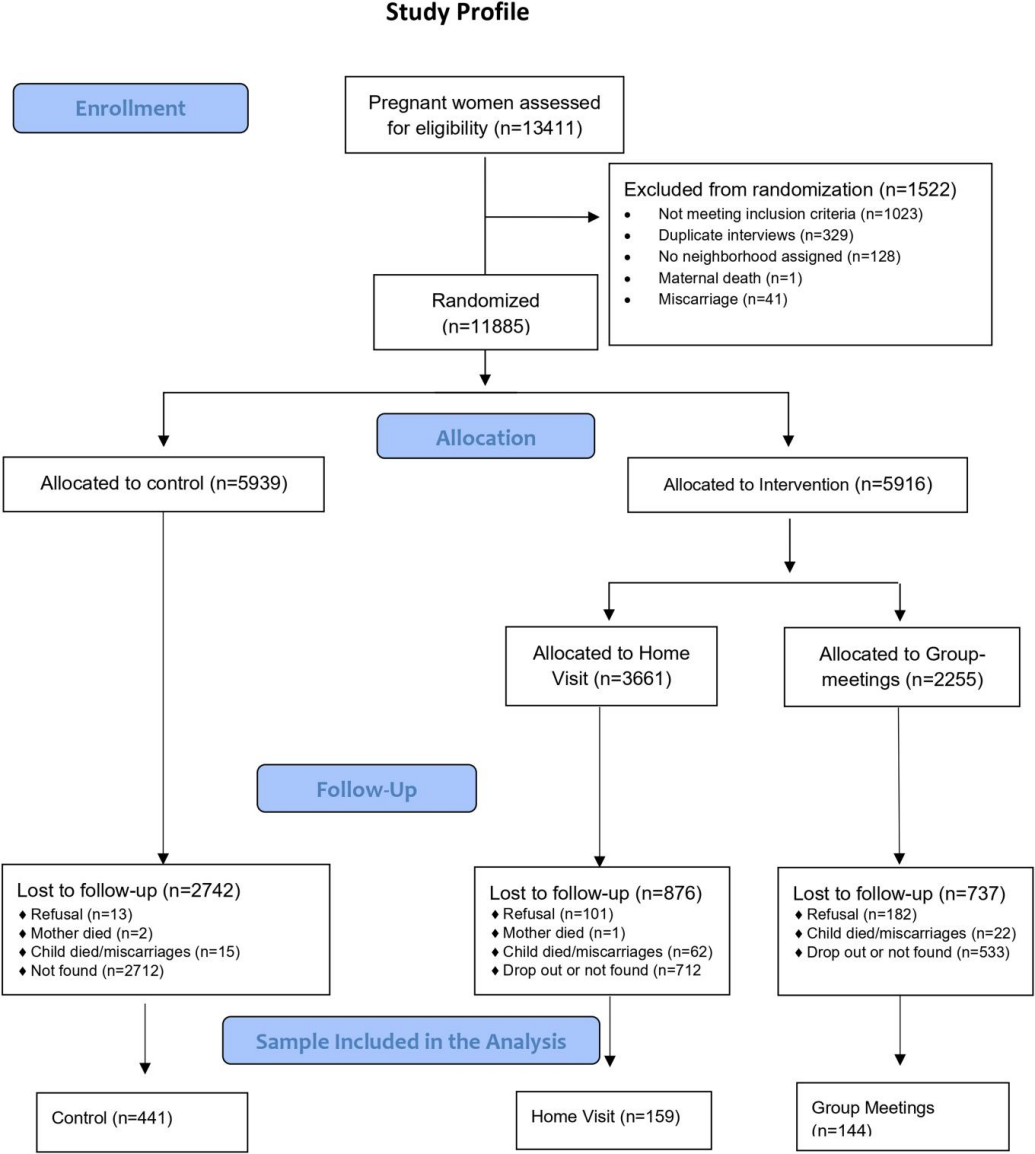
Trial profile.

Figure 2 shows the participants’ flow.

Over the study period, 13 411 pregnant women were assessed for eligibility, 1522 were excluded from randomization because they did not meet the inclusion criteria, were interviewed multiple times, had no neighborhood information, or died/miscarried before randomization. A total of 5939 pregnant women were allocated to the control group, and 5916 were allocated to one of the intervention groups (3661 were allocated to the HV study group, and 2255 were allocated to group meetings). In the HV arm, 101 (2.76%) pregnant women refused to be enrolled in the program, and 3459 (94.5%) were enrolled and started receiving HVs (471 in Year 1, 1443 in Year 2, and 1646 in Year 3). During the study period 1 mother died, 62 miscarried, and 712 (20.6%) discontinued their participation (voluntarily or because they changed address and could not be found). In the group meeting arm, 182 (8.1%) pregnant women refused enrollment in the program, and 2073 (92%) were enrolled and started participating in group meetings. Twenty-two discontinued participations due to miscarriage or the child’s death, and 533 (26%) discontinued voluntarily or because they changed address and could not be found.

A total of 744 children were assessed at their homes at endline. SDQ assessments were completed for 743 children (99%), PRIDI assessments for 681 children (92%), and CREDI assessments for 545 children (73%). Table 1 summarizes sample characteristics by study group. Five percent of caregivers were under age 20—the large majority (71%) were between ages 20 and 34. Fifty-seven percent of mothers had completed secondary schooling, and 9% had attained higher education. Thirteen percent of caregivers were Venezuelan and 10% had other nationalities. Most families had a TV (81%), and only 14% had a car. On average, children were 30 months old when the CREDI assessment was done, and 34 months old when the PRIDI and SDQ assessments were done. Caregivers in the CB group were slightly younger and less educated than caregivers in the HV group—none of these differences were statistically significant. The difference in mean age between the HM and CB groups is explained by the later launch of the CB intervention as described before. Average ages for the CREDI are younger (and sample sizes are lower) because the CREDI tool was only administered to children under age 3 as recommended.

**Table 1:** Sample characteristics by treatment group.

	Stats	Control		CB		HVs	
		*N* = 441		*N* = 144		*N* = 159	
Caregiver under age 20	N, %	19	4.3%	9	6.3%	8	5.0%
Caregiver age 20–34	N, %	300	68.0%	113	78.5%	118	74.2%
Caregiver age 20–34	N, %	79	17.9%	14	9.7%	24	15.1%
Caregiver age 35–39	N, %	43	9.8%	8	5.6%	9	5.7%
Caregiver not mother	N, %	32	7.3%	8	5.6%	6	3.8%
Mother: no schooling	N, %	6	1.4%	10	6.9%	6	3.8%
Mother: basic schooling	N, %	130	29.5%	48	33.3%	46	28.9%
Mother: secondary	N, %	265	60.1%	72	50.0%	86	54.1%
Mother: higher education	N, %	35	7.9%	13	9.0%	20	12.6%
Married or living with partner	N, %	280	63.5%	91	63.2%	99	62.3%
Mother consumes alcohol	N, %	20	4.5%	4	2.8%	4	2.5%
Mother smokes	N, %	9	2.0%	5	3.5%	2	1.3%
Mother Brazilian	N, %	329	74.6%	120	83.3%	124	78.0%
Mother Venezuelan	N, %	57	12.9%	20	13.9%	20	12.6%
Mother other nationality	N, %	55	12.5%	4	2.8%	15	9.4%
Family receives Bolsa Familia	N, %	99	22.4%	37	25.7%	24	15.1%
Household has TV	N, %	358	81.2%	113	78.5%	128	80.5%
Household has car	N, %	62	14.1%	17	11.8%	26	16.4%
Home quality score (1–10)	Mean, SD	5.9	1.9	6.2	2.1	5.9	2.1
Child age at PRIDI assessment	Mean, SD	36.8	5.1	30.8	5.3	31.1	5.1
Child age at CREDI assessment	Mean, SD	30.8	3.2	28.0	2.7	28.8	2.5

Table 2 shows the estimated impact on child development. In adjusted models, HVs increased average PRIDI scores by about 0.3 SDs. For CREDI scores, estimated impacts were significant in the RE models, but smaller and nonsignificant in the FE models. No significant impact was found on the SDQ (lower scores show fewer behavior difficulties). For CB programming, no significant impacts were found in the adjusted estimates. Although the estimated difference for the PRIDI in the FE model was similar to that for HV, it was not statistically significant.

**Table 2:** Impact on child development.

Outcome	PRIDI		CREDI		SDQ	
	(1)	(2)	(3)	(4)	(5)	(6)
Model	RE	FE	RE	FE	RE	FE
Unadjusted estimates						
CB	−0.178**	0.187	−0.0295	0.398	0.00471	−1.076
	(−0.354—−0.00296)	(−0.246–0.620)	(−0.313–0.254)	(−0.427–1.223)	(−1.155–1.164)	(−3.126–0.975)
HV	0.130	0.280*	0.378***	−0.0791	−0.220	−0.587
	(−0.0397–0.299)	(−0.0463–0.607)	(0.105–0.651)	(−0.662–0.504)	(−1.332–0.892)	(−2.385–1.211)
Observations	681	681	545	545	743	743
Adjusted estimates						
CB	0.0500	0.250	−0.0622	0.299	−0.0813	−1.139
	(−0.125–0.225)	(−0.163–0.662)	(−0.351–0.227)	(−0.505–1.103)	(−1.327–1.164)	(−2.844–0.566)
HV	0.240***	0.278*	0.287**	−0.158	−0.191	−0.564
	(0.0712–0.409)	(−0.0125–0.568)	(0.0132–0.561)	(−0.709–0.392)	(−1.389–1.006)	(−2.480–1.352)
Observations	673	673	540	540	735	735

Results presented by RE and FE models. Robust standard errors in parentheses are clustered at the neighborhood (bairro) level. All coefficients are z-scores.

***P < .001; **P < .05; *P < .1

**Figure 3:**
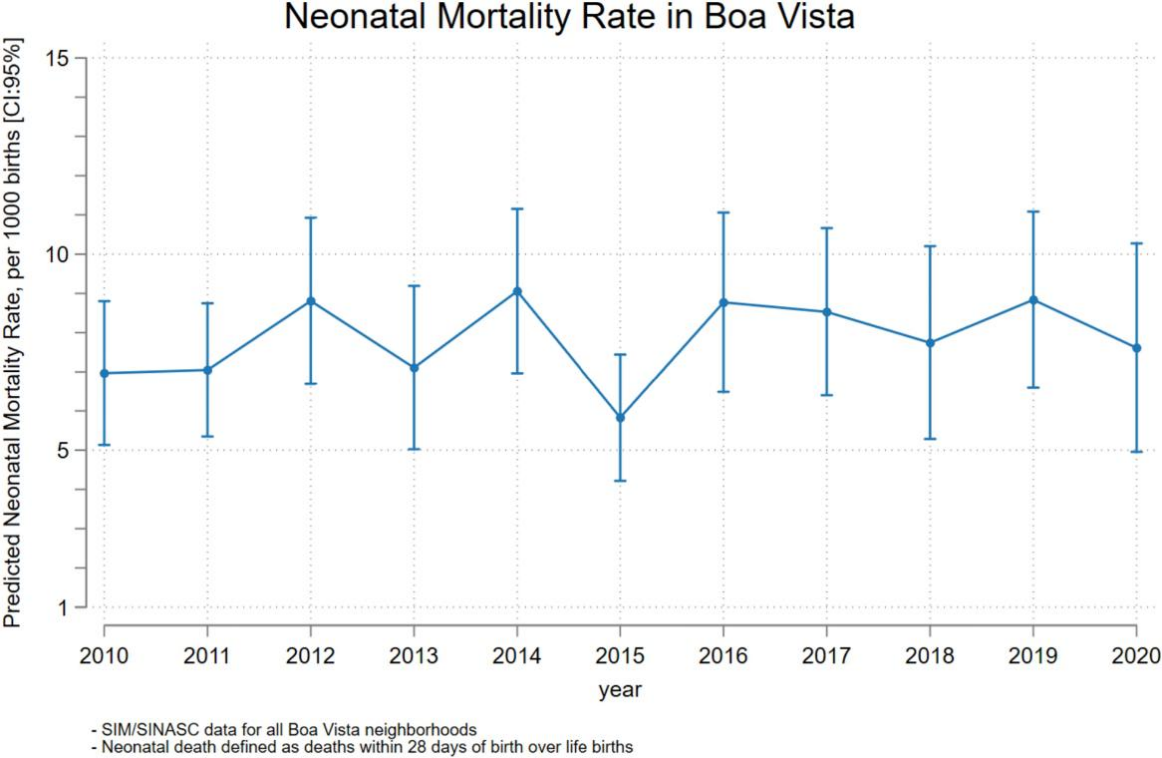
Neonatal mortality rate in Boa Vista over mortality period. SIM/SINASC data for all Boa Vista neighborhoods. Neonatal death defined as deaths within 28 days of birth over live births.

The neonatal survival analysis data set comprised 77 310 live births and 647 neonatal deaths (535 early neonatal deaths). Starting from 2010, a steady increase in the number of births was observed, more markedly so from 2016 to 2019 (see Supplementary Figure S4a). Annual neonatal deaths in Boa Vista (from 2010 to 2020) ranged from 39 to 79 (see Supplementary Fig. S4b). Figure 3 shows neonatal mortality rate (NNMR) per 1000 live births in the study period—NNMR fluctuated between six and nine deaths per 1000, with an average level of 8.3 deaths across the full sample period.

The Intention to Treat (ITT) regression models estimated the effect of HV or CB treatment on neonatal mortality, with Risk Ratio (RR) of 0.58 and 0.95 and 95% Confidence Interval (CI) of 0.36–0.93 and 0.61–1.46, respectively (Table 3). The impact of HV on early neonatal mortality was weaker and nonsignificant. Unadjusted models and models using only complete cases (data not shown) produced very similar results.

**Table 3:** Treatment effects of full HV and CB treatment on neonatal mortality.

	Neonatal mortality crude			Neonatal mortality adjusted			Early neonatal mortality crude			Early neonatal mortality adjusted			Live births	Neonatal deaths	Early neonatal deaths
	RR	CI95	*P*-value	RR	CI95	*P*-value	RR	CI95	*P*-value	RR	CI95	*P*-value	*N* = 77 310	*N* = 642	*N* = 535
Full treatment															
Control	1.00		.	1.00		.	1.00		.	1.00		.	65 567	562	456
HV	0.57	(0.35–0.91)	.020	0.49	(0.31–0.77)	.002	0.69	(0.41–1.15)	.152	0.60	(0.37–0.98)	.041	7220	45	41
CB	0.93	(0.60–1.44)	.748	0.82	(0.51–1.31)	.401	1.17	(0.75–1.83)	.497	1.07	(0.67–1.72)	.769	3876	40	38

Results are based on generalized linear models including partial initial and full treatment periods, year, and neighborhood clustered standard errors. Crude models are based on the full imputed mortality analysis set (*N* = 77 310); adjusted models additionally include maternal age, maternal education, mother’s nationality, birth mode, and a multiplicative bairro–year interaction term. Adjusted neonatal mortality model dropped four bairros (*N* = 395) due to absence of deaths. Total model *N* = 76 915 with live births Ns: control = 65 266 HV = 7126; CB = 3876. Adjusted early neonatal mortality model dropped five bairros (*N* = 681) due to absence of deaths. Total model *N* = 76 629 with live births Ns: control = 65 104; HV = 7112; CB = 3878.

## Discussion

The results presented here suggest that the modified Reach Up intervention package had positive impacts on child survival and child development. The effect size of the impacts on child development is comparable to previous Reach Up–based studies (Jervis et al. 2023) and shows a larger impact than a previous intervention study in São Paulo, which found positive treatment effects only among compliers (Brentani et al. 2021). These positive developmental impacts also contrast with the recent national evaluation of the PCF, which found no impact on child development (Goncalves et al. 2019, Santos et al. 2022, Avaliação de impacto do programa criança feliz em saúde materno-infantil 2023).

Compared to most interventions utilized in PCF, the intervention evaluated here offers a structured curriculum with age-appropriate activities for each visit, comprehensive initial training, and detailed supervision to ensure visit quality (Filene et al. 2018). These components appear to have worked well with HVs. The main challenge with the CB program was caregiver participation. In the context of our study, we found that only 12% of caregivers attended more than five sessions, and <5% of caregivers attended more than 10. Although group meetings were organized through social assistance units (and thus should have been reachable for all), qualitative data collected as part of this study suggests that some participants still had challenges related to transportation costs, while others could not leave their other children unattended at home to participate in the meetings. Additionally, during group meetings, fewer activities were introduced (two activities per meeting instead of the three or four per HV), which may have contributed to the lack of impact.

The most remarkable results of this study are the improvements in neonatal survival. At the outset of this program, reducing neonatal mortality was declared a primary goal by the municipality, and pregnancy and neonatal modules were added to the curriculum to provide support for vulnerable families starting from the third trimester, hoping to see treatment effects seen in previous related studies (Bhutta et al. 2011). Most of the benefits were observed after the early neonatal period, when caregiver behavior is likely more important than during the period immediately after birth (which mostly happens at hospitals in this setting) (Prezotto et al. 2023). Many mothers did not attend the CB group sessions held during the first and third weeks after birth, and there was no impact on neonatal mortality in the CB arm.

The study has several limitations. The COVID-19 pandemic restricted face-to-face visits. During this period, the intervention’s reach was limited to only families who could be contacted online (via WhatsApp), thus reducing the expected exposure to the intervention. To address this, we focused our endline sample on children enrolled in 2019 who received most visits face-to-face. This resulted in a smaller child development sample size than originally planned and limited our ability to do subgroup analysis. Moreover, many children were older than 36 months at assessment, limiting the CREDI assessment to a partial sample at endline. Additionally, the CB group meeting sessions were launched 6 months after the HVs due to administrative issues, which accounted for the difference in child age between the study arms.

Despite these limitations, we found significant improvements in child development. We used two child development assessment instruments: one caregiver-reported (CREDI) and one directly administered (PRIDI), which supports the robustness of our findings.

The random rollout of the program at the neighborhood level allowed us to minimize contamination concerns. At the same time, we observed substantial heterogeneity with respect to program reach, with coverage rates ranging between 25% and 95% across neighborhoods.

This study demonstrating a positive impact on child development and neonatal mortality reduction offers valuable insights for the PCF and for the continued implementation of this national ECD home visiting program in Brazil and for other similar programs globally. Our results also show that combining antenatal and neonatal modules with the Reach Up curriculum has the potential to help children to survive and thrive during early childhood.

CB programs, with small groups of mothers and children, can potentially be more cost-effective and have been shown to benefit child development in other settings (Grantham-McGregor et al. 2020), but in this study program, attendance for the CB model was low. Measures to increase parental attendance need to be tested and adopted for effective CB delivery to be achieved.

## Conclusion

Results presented here suggest that HVs that combine pregnancy and neonatal modules with a Reach Up–based curriculum can be effective in helping vulnerable children survive and thrive. Further work is needed to develop effective CB delivery in this context.

## Supplementary Material

czag057_Supplementary_Data

## Data Availability

The data underlying this article will be shared on reasonable request to the corresponding author.

## Reflexivity statement

The authors include seven females and two males and span multiple levels of seniority. While one of the authors specialize in child development, HV programs, and health policy in Brazil, three authors are pediatricians from Brazil, three authors are economists specialized in global health, and two authors are representatives from the original Reach Up group from Jamaica. Six authors have extensive experience conducting randomized controlled trials, especially in Brazil, where a previous version of the project was implemented.
