# Supplementary material for: Survive and Thrive in Brazil: estimating the impact of the Boa Vista early childhood program
Source: Health Policy Plan. 2026 Apr 27;41(7):1115–25. doi: 10.1093/heapol/czag057 (PMC13481676; doi:10.1093/heapol/czag057)
Supplement: Supplementary file 1 [file czag057_supplementary_data.docx]

**SUPPLEMENTARY MATERIALS**

**Methods**

**Probabilistic matching**

SIM neonatal deaths and SINASC live birth records were merged creating the analysis data set as follows. First, the majority of SIM neonatal deaths contained the SINASC unique identifier and were merged directly with SINASC. The remainder was matched to SINASC using probabilistic matching on mothers’ names, residential neighborhood, and child’s birth year, and each match was checked manually for appropriate matching to SINASC. The remaining neonatal deaths, not matched via unique SINASC ID or probabilistic matching above, were matched manually using additional information (child name or father name) when unambiguously possible. From 2010 to 2020 inclusive, SIM recorded 663 neonatal deaths in the Boa Vista municipality. We matched 649 (97.9%) with their corresponding SINASC live births records, i.e. 531 (81.8%) were matched via SINASC ID, 77 (11.7%) were matched probabilistically, and 34 (5.2%) were matched manually. Out of the remaining 21 (3.2%) not matched, 7 (1.1%) had complete residence and birth date information and were included in the analysis set, assuming these births were not recorded in SINASC at all. 8 (1.2%) records missed residential information (thus not usable to assign treatment) and 6 (0.9%) were matched to mothers outside of Boa Vista and were not included in the analysis. The SINASCS total number of births in Boa Vista neighborhoods participating in the Survive and Thrive trial from 2010-2020 inclusive was 77,518. In the SIM and SINASC merged data set, 208 records did not provide residential neighborhood information in SIM or SINASC and had to be omitted, resulting in 77,310 (99.7%) live births and 647 (99.7%) neonatal deaths used in the multiple imputation analysis (and 77,207 and 643 respectively in the complete cases analysis).

Figure s1: Location of Boa Vista Municipality

| Panel A: Roraima State within Brazil | Panel B: Boa Vista Municipality within Roraima |
| --- | --- |
|  |  |

Figure s2: Neighborhood vulnerability index

Figure s3A: Intervention coverage periods of Home visit Treatment group by neighborhood; Grey lines represent starting period of 90 days, colored lines full intervention phase

Figure s3B: Intervention coverage periods of Center Based treatment group by neighborhood; Grey lines represent starting period of 90 days, colored lines full intervention phase; neighborhoods without intervention are detailed at the bottom

Figure s4A: Annual numbers of live births in Boa Vista

Figure s4B: Annual number of neonatal deaths in Boa Vista

***Supplementary Materials Table s1: Number of children per study arm with available child development data***

| **Bairro** | **Total** | **Controls** | **Center-based** | **Home visits** |
| --- | --- | --- | --- | --- |
| 13 DE SETEMBRO | 13 | 13 | 0 | 0 |
| AEROPORTO | 7 | 0 | 7 | 0 |
| ALVORADA | 8 | 2 | 0 | 6 |
| APARECIDA | 1 | 1 | 0 | 0 |
| ARACELI SOUTO MAIOR | 21 | 4 | 17 | 0 |
| ASA BRANCA | 36 | 36 | 0 | 0 |
| BELA VISTA | 7 | 0 | 7 | 0 |
| BURITIS | 23 | 9 | 0 | 14 |
| CAIMBE | 21 | 21 | 0 | 0 |
| CALUNGA | 3 | 1 | 0 | 2 |
| CAMBARA | 2 | 0 | 0 | 2 |
| CARANA | 42 | 42 | 0 | 0 |
| CAUAME | 19 | 12 | 0 | 7 |
| CENTENARIO | 4 | 0 | 0 | 4 |
| CENTRO | 6 | 0 | 6 | 0 |
| CIDADE SATELITE | 95 | 95 | 0 | 0 |
| CINTURAO VERDE | 7 | 7 | 0 | 0 |
| DOS ESTADOS | 1 | 1 | 0 | 0 |
| DOUTOR AIRTON ROCHA | 14 | 0 | 0 | 14 |
| DOUTOR SILVIO BOTELHO | 13 | 9 | 0 | 4 |
| DOUTOR SILVIO LEITE | 21 | 0 | 21 | 0 |
| EQUATORIAL | 13 | 0 | 0 | 13 |
| GOV AQUILINO MOTA DUARTE | 1 | 0 | 0 | 1 |
| JARDIM CARANA | 5 | 0 | 0 | 5 |
| JARDIM FLORESTA | 7 | 3 | 4 | 0 |
| JARDIM PRIMAVERA | 5 | 0 | 5 | 0 |
| JARDIM TROPICAL | 8 | 0 | 8 | 0 |
| JOQUEI CLUBE | 10 | 4 | 6 | 0 |
| LAURA MOREIRA | 34 | 18 | 16 | 0 |
| LIBERDADE | 8 | 2 | 0 | 6 |
| MECEJANA | 4 | 4 | 0 | 0 |
| NOVA CANAA | 2 | 0 | 0 | 2 |
| NOVA CIDADE | 50 | 50 | 0 | 0 |
| OLIMPICO | 6 | 0 | 0 | 6 |
| OPERARIO | 3 | 0 | 0 | 3 |
| PINTOLANDIA | 11 | 0 | 11 | 0 |
| PRICUMA | 12 | 12 | 0 | 0 |
| RAIAR DO SOL | 15 | 8 | 0 | 7 |
| SAID SALOMAO | 7 | 0 | 0 | 7 |
| SANTA LUZIA | 6 | 0 | 6 | 0 |
| SANTA TEREZA | 10 | 2 | 8 | 0 |
| SAO BENTO | 14 | 4 | 0 | 10 |
| SAO FRANCISCO | 1 | 1 | 0 | 0 |
| SAO VICENTE | 23 | 23 | 0 | 0 |
| SENADOR HELIO CAMPOS | 41 | 17 | 0 | 24 |
| TANCREDO NEVES | 14 | 11 | 0 | 3 |
| UNIAO | 7 | 0 | 7 | 0 |

***Table s2: Mortality Analysis missingness, addressed with multiple imputations***
